# *In Vitro* Culture with Interleukin-15 Leads to Expression of Activating Receptors and Recovery of Natural Killer Cell Function in Acute Myeloid Leukemia Patients

**DOI:** 10.3389/fimmu.2017.00931

**Published:** 2017-08-07

**Authors:** Beatriz Sanchez-Correa, Juan M. Bergua, Alejandra Pera, Carmen Campos, Maria Jose Arcos, Helena Bañas, Esther Duran, Rafael Solana, Raquel Tarazona

**Affiliations:** ^1^Immunology Unit, University of Extremadura, Cáceres, Spain; ^2^Department of Haematology, Hospital San Pedro de Alcantara, Cáceres, Spain; ^3^Instituto Maimónides de Investigación Biomédica de Córdoba (IMIBIC), Reina Sofia University Hospital, University of Cordoba, Red Espanola de Investigacion en Patologia Infecciosa (REIPI), Córdoba, Spain; ^4^Brighton and Sussex Medical School, University of Sussex, Brighton, United Kingdom; ^5^Histology and Pathology Unit, Faculty of Veterinary, University of Extremadura, Cáceres, Spain

**Keywords:** natural killer cell, acute myeloid leukemia, interleukin-15, NKp30, dendritic cells, natural killer cell degranulation

## Abstract

Despite recent progress in the therapeutic approach of malignant hemopathies, their prognoses remain frequently poor. Immunotherapy could open a new window of great interest in this setting. Natural killer (NK) cells constitute an important area of research for hematologic malignancies, because this subpopulation is able to kill target cells spontaneously without previous sensitization, representing a novel tool in the treatment of them. Abnormal NK cytolytic function is observed in several hematological malignancies, including acute myeloid leukemia (AML) and myelodysplastic syndromes. Several mechanisms are involved in this abnormal function, such as decreased expression of activating receptors, increased expression of inhibitory receptors or defective expression of NK cell ligands on target cells. New immunotherapies are focused in identifying factors that could increase the expression of these activating receptors, to counteract inhibitory receptors expression, and therefore, to improve the NK cell cytotoxic capacities against tumor cells. In this work, we analyze the effect of interleukin (IL)-15 on the expression of NK cell-activating receptors that play a crucial role in the lysis of blasts from AML patients. Our results showed that IL-15 increased the surface expression of NKp30 on NK cells from healthy donors and AML patients with the consequent improvement of NK cell cytotoxicity. Besides, the upregulation of NKp30 induced by IL-15 is associated with an improvement of NK-mediated myeloid dendritic cells (DCs) maturation. NK cells cultured with IL-15 showed an upregulation of NKp30, which is associated with an increase anti-tumor activity and with an improved maturation of immature DCs. In our *in vitro* model, IL-15 exerted a great activating stimulus that could be used as novel immunotherapy in AML patients.

## Introduction

Natural killer (NK) cells constitutively possess lytic machinery and are able to spontaneously lyse virally infected or malignant cells without any prior sensitization to antigen. Their function is tightly controlled through a balance of signals from activating and inhibitory receptors, by cytokines and chemokines (1–3), as well as by cross talk with other immune cells, such as dendritic cells (DCs) (4), effector CD4^+^ T cells (5), and regulatory T cells (Tregs) (6, 7).

There are several major histocompatibility complex (MHC) class I-specific inhibitory receptors with different molecular structures and specificities for different alleles of human leukocyte antigen (HLA) class I molecules. These receptors act as negative regulators of NK cytotoxicity ensuring that normal autologous cells are preserved (4, 8, 9). The two main groups of inhibitory receptors are the killer Ig-like receptors (KIR) (8), which recognize polymorphic determinants of HLA-A, -B, or -C molecules and the heterodimeric receptors CD94-NKG2A/B, which recognize peptides derived from the leader sequences of different HLA class I molecules presented by the non-classical MHC class I molecule HLA-E (10–13). Normal autologous cells that express HLA class I molecules are protected from NK-mediated attack, but cells that have reduced expression because of malignant transformation become susceptible to NK cell attack. Loss or downregulation of a single HLA-I allele, a relatively frequent event in cancer, can be sufficient to make tumor cells susceptible to NK cell cytotoxicity (8, 9).

In the absence of inhibitory signals, NK cell cytotoxicity requires signaling through activating receptors upon interaction with their ligands on target cells. An ample set of activating receptors have been described including, among others, members of the C lectin-like family as NKG2D and members of the immunoglobulin superfamily as natural cytotoxicity receptors (NCRs) and DNAM-1 (also known as CD226) (4, 8, 9, 14). NKG2D ligands are a group of MHC class I-like molecules, the expression of which is induced by cellular stress. NKG2D ligands included MHC-I polypeptide-related sequences A (MICA) and B (MICB), and UL16-binding proteins (ULBP1–6) (15, 16). DNAM-1 specifically recognizes CD155 and CD112, two members of the nectin family, that are also expressed on different types of tumors (17–20).

Natural cytotoxicity receptors are major activating receptors involved in tumor cell detection and lysis. NCRs include NKp46 (21–23), NKp30 (24, 25), and NKp44 (26), which mediate cell lysis of many cancer cells. NKp30 and NKp46 are expressed both in resting and activated NK cells, whereas NKp44 expression is restricted to activated NK cells (4, 9, 14, 21, 27). NCR ligands include pathogen-associated as well as stress-related molecules. The identity of NCR ligands on tumors remains in part elusive. The nuclear factor HLA-B-associated transcript 3 (BAT3) and B7H6 have been described as NKp30 ligands (24, 28, 29). NKp46 and NKp44 were shown to interact with the viral hemagglutinin protein (24, 30–32). A truncated isoform of mixed lineage leukemia 5 is an activating ligand for NKp44 (33). By contrast, proliferating cell nuclear antigen is an inhibitory tumor ligand for NKp44 (34). No tumor ligands for NKp46 have been identified so far.

We have recently demonstrated that acute myeloid leukemia (AML) patients have depressed NK cell function as well as altered cytokine production (35–37). NK cell-mediated rejection of leukemic blasts may be limited by the reduced expression of NK cell-activating receptors such as DNAM-1, NKp46, and NKp30 observed in AML patients (36, 37). Besides, NKp46 downregulation has been associated with decreased survival in AML patients (38).

In the last years, several cytokines have been extensively studied as potential therapeutic agents to manipulate the immune response against malignant cells due to their capacity of stimulate cell growth and survival as well as increase the cytotoxicity or cytokine production to boost immune reactivity (39–42). So far, only a small number of cytokines have reached clinical use probably due to the complexity of cytokine network. Among these cytokines tested in different *in vitro* and *in vivo* settings, interleukin (IL)-2 and IL-15 should be highlighted (40, 41, 43, 44). IL-2, initially described as a T cell growth factor, promotes CD8^+^ T cell and NK cell cytolytic activity and modulates T cell differentiation in response to antigen. Moreover, IL-2 is essential for the development and maintenance of Tregs that may represent a limitation for its use in patients with cancer. The major disadvantage of IL-2 is its toxicity, including severe capillary leak syndrome that can accompany this treatment (43). Recently, IL-15 has emerged as a potential immunotherapeutic candidate for the treatment of cancer. IL-2 and IL-15 are structurally related and have overlapping functions including their role in T cell proliferation, promotion of cytotoxic T cell differentiation, production of immunoglobulin by B cells, and generation, proliferation, and activation of NK cells. In contrast to IL-2, IL-15 is not required for the maintenance of Tregs and, based on preclinical studies, IL-15 causes less vascular capillary leak (42). These factors support the role of IL-15 for cancer immunotherapy, boosting both innate and adaptive immunity against tumors (45). However, so far, few clinical trials have analyzed the security and efficacy of IL-15 in cancer patients. Thus, a Phase I study (NCT01572493) assessing the safety and efficacy of IL-15 in adults with advanced malignancies has been suspended for undisclosed reasons. IL-15 is being tested as an immunological adjuvant to haploidentical NK cell transfer in AML patients (NCT02395822).

Due to the ability of NK cells to spontaneously kill tumor cells, this population represents an attractive tool for cancer immunotherapy (46–48). However, NK cell defective function in AML patients may limit tumor control. The possibility of manipulating NK cells by cytokines for therapeutic purposes open new area of research in cancer.

The biological effects of IL-15 on NK cells will depend on the direct effect on NK cells as well as by indirect consequences mediated by other cells stimulated by IL-15. Thus, in order to analyze the whole figure we have used peripheral blood mononuclear cell (PBMC) cultures stimulated with IL-15.

In this study, we aimed to assess (i) whether IL-15 induces and expansion of NK cells from healthy donors (HDs) and AML patients and its effect on the expression of activating receptors after short-term culture *in vitro*; (ii) whether IL-15 increases the cytotoxic activity of NK cells; and (iii) whether the maturation of immature DCs (iDCs) is enhanced by culture with IL-15-stimulated PBMCs.

## Materials and Methods

### Patients and HDs

Peripheral blood mononuclear cells were obtained from 14 newly diagnosed AML patients (ranged 18–89 years) at the Hospital San Pedro de Alcántara (Cáceres, Spain) prior to any treatment and from 20 HD volunteers (ranged 20–60 years). The study was approved by the local Ethics Committee and samples collected after written informed consent in accordance with the Declaration of Helsinki. Diagnosis was established by cytological criteria based on the French–American–British classification.

The collected blood was drawn into heparinized tubes and processed using Ficoll-Hypaque gradients. PBMCs were recovered and cell count and viability analysis were performed. PBMCs were immediately used for experiments. For co-culture experiments, we selected those patients with lower percentage of leukemic blasts and to eliminate leukemic blasts an adherence step was included in the protocol prior to co-culture with DCs.

Plasma was obtained after centrifugation and stored at −80°C for measurement of cytokine levels. Plasma samples from HDs and from AML patients used in the study had not been previously thawed.

### Monoclonal Antibodies (mAbs)

Natural killer cell percentage and phenotype were evaluated in PBMC obtained from AML patients and HDs. The following anti-human mAbs were used for flow cytometry: CD56-FITC (NCAM16.2), CD56-PE (MY31), CD14-PE (MØP9), CD3-PerCP (SK7) all from BD Biosciences (San Jose, CA, USA); CD56-PECy7 (B159), CD16-APC Cy7 (3G8), NKG2D-PE (1D11), CD226-PE (DX11), CD107a-FITC (H4A3), CD107b-FITC (H4B4), CD86-FITC (2331(FUN-1)), CD1a-FITC (HI149) all from BD Pharmingen (San Diego, CA, USA); and NKp30-PE (AF29-4D12), NKp30-APC (AF29-4D12), NKp46-PE (9E2), NKp46-APC (9E2), CD3-VioBlue (BW264/56) all from Miltenyi Biotec (Bergisch Gladbach, Germany). Prior to use, mAbs were titrated to establish optimal staining dilutions. Isotype-matched immunoglobulins were included in all experiments as negative controls. Mean relative fluorescence intensity was calculated by dividing the mean fluorescent intensity (MFI) of the relevant mAb by the MFI of its isotype control.

### Cell Culture and Flow Cytometry Analyses of NK Cells

Peripheral blood mononuclear cells were cultured in complete medium (RPMI-1640 supplemented with 10% FCS, l-glutamine, sodium pyruvate, non-essential amino acids and penicillin/streptomycin, all from BioWhittaker, Verviers, Belgium) and were stimulated with 100 ng/mL of recombinant human (rh)IL-15 from Peprotech (Rocky Hill, NJ, USA) or 750 U/mL of rhIL-2 (National Cancer Institute, Frederick, MD, USA) (49). Cells were harvested after 48 h, and the frequency and NK receptor repertoire were assessed by multi-parameter flow cytometry using a FACScan cytometer and the CellQuest software (BD Biosciences) or MACsQuant cytometer and the MACSQuantify software (Miltenyi Biotec). NK cells were defined as CD3^−^CD56^+^ cells within the lymphocyte gate and the expression of activating receptors analyzed was referred to this population. Analysis of NK cells at various time points after cytokine activation was performed in order to select the best timing for functional analysis (data not shown).

### Analysis of IL-15 in Plasma

Interleukin-15 concentrations in the plasma of patients and healthy controls were determined by enzyme-linked immunosorbent assay (ELISA) using a Human IL-15 ELISA Ready-SET-Go! Kit (eBioscience) according to the manufacturer’s instructions. The minimum detectable levels were 8 pg/mL, and the standard curve range was 8–1,000 pg/mL.

Two independent sets of experiments were performed. No significant variations were observed among the experiments. Plate was read in an Infinite^®^ 200 (Tecan, Switzerland) plate reader.

### NK Cell Degranulation Assay

Cytokine-stimulated NK cells were tested in a degranulation assay against the NK cell-susceptible target cell line K562. The analysis of NK cell degranulation was performed by measuring the expression of CD107a/b after activation with target cells at ratio 1:1 in the presence of BD GolgiStop (BD Biosciences) and a mixture of FITC-labeled anti-CD107a and anti-CD107b mAbs. After 4 h, cells were stained with PE-labeled anti-CD56 and PercP-labeled anti-CD3 from BD Biosciences and analyzed by flow cytometry by measuring the frequency of CD107a/b expression on CD3^−^CD56^+^ NK cells. Spontaneous basal NK cell degranulation was always below 10%. Background expression of CD107a/b (CD107a^+^ NK cells in medium only) was subtracted from expression with target cells.

### Generation of DCs

Monocyte-derived iDCs were obtained by adherence of monocytes to plastic. Thus, PBMCs from HDs were resuspended at 5 × 10^6^ cells/mL in complete medium and allowed to adhere for 2 h at 37°C in culture flasks. Then, the non-adherent cells were removed and the adherent cells, predominantly monocytes, were cultivated in complete medium supplemented with 50 ng/mL of recombinant human granulocyte-macrophage colony-stimulating factor (Peprotech) and 20 ng/mL of rhIL-4 (Peprotech). After 6 days, the percentage of iDCs was analyzed by flow cytometry. iDCs were defined as CD14^−^CD1a^+^CD83^−^CD86^−^ cells.

To generate mDCs, iDCs were plated either in the absence or in the presence of allogeneic PBMCs cells stimulated or not with rhIL15 or rhIL-2 at ratio 1:5. After 2 days, DCs were assessed for the expression of CD86. As positive control, optimal DC maturation was induced by *Escherichia coli* lipopolysaccharide (LPS) 1 µg/mL (serotype 055:B5, Sigma-Aldrich, St Louis, MO, USA).

### Statistical Analysis

Statistical analysis was performed using SPSS Statistics version 19. Because the cell population counts were generally not normally distributed, we report medians and ranges for parameter values. Non-parametric statistical methods were used to analyze the data. Paired differences between NK cells from the same patient with or without stimulation by cytokines were tested using the Wilcoxon signed-rank test. For comparison of NK cells between HDs and AML patients, the exact Wilcoxon rank sum test was used.

## Results

### Effect of IL-2 and IL-15 on NK Cell Expansion *In Vitro*

**Figure 1:**
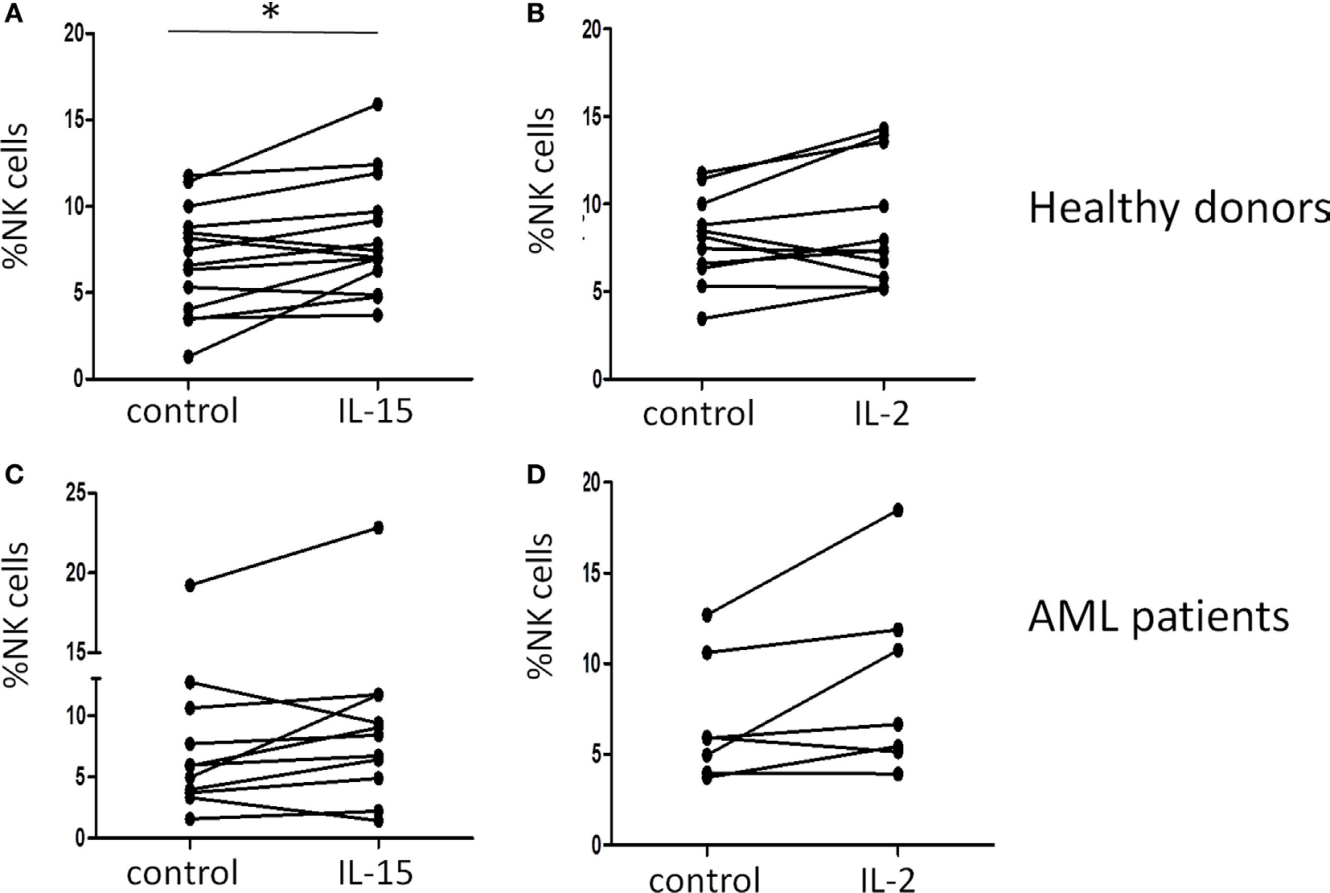
Effect of interleukin (IL)-15 and IL-2 on natural killer (NK) cell expansion *in vitro*. The increase in the percentage of NK cells after incubation for 48 h with cytokines was analyzed by flow cytometry in healthy donors **(A,B)** and acute myeloid leukemia (AML) patients **(C,D)** in the presence of IL-15 **(A,C)** or IL-2 **(B,D)**.

Natural killer cells were evaluated in HDs and AML patients at the time of diagnosis and prior to any treatment. In order to study the modulatory effect of IL-2 and IL-15 on NK cell proliferation, we cultured PBMCs from HDs and AML patients in presence of these cytokines. Our results showed that IL-15 but not IL-2 induced a significant increase (*p* = 0.016) in the percentage of NK cells from HDs after 48 h of culture (Figures 1A,B). By contrast, none of the cytokines had an effect on the percentage of NK cells from AML patients (Figures 1C,D).

### Upregulation of NK Cell-Activating Receptors by IL-2 and IL-15 *In Vitro*

In order to assess the effect of IL-2 and IL-15 on the NK cell receptor expression, we cultured PBMCs from HDs and AML patients in presence of these cytokines.

**Figure 2:**
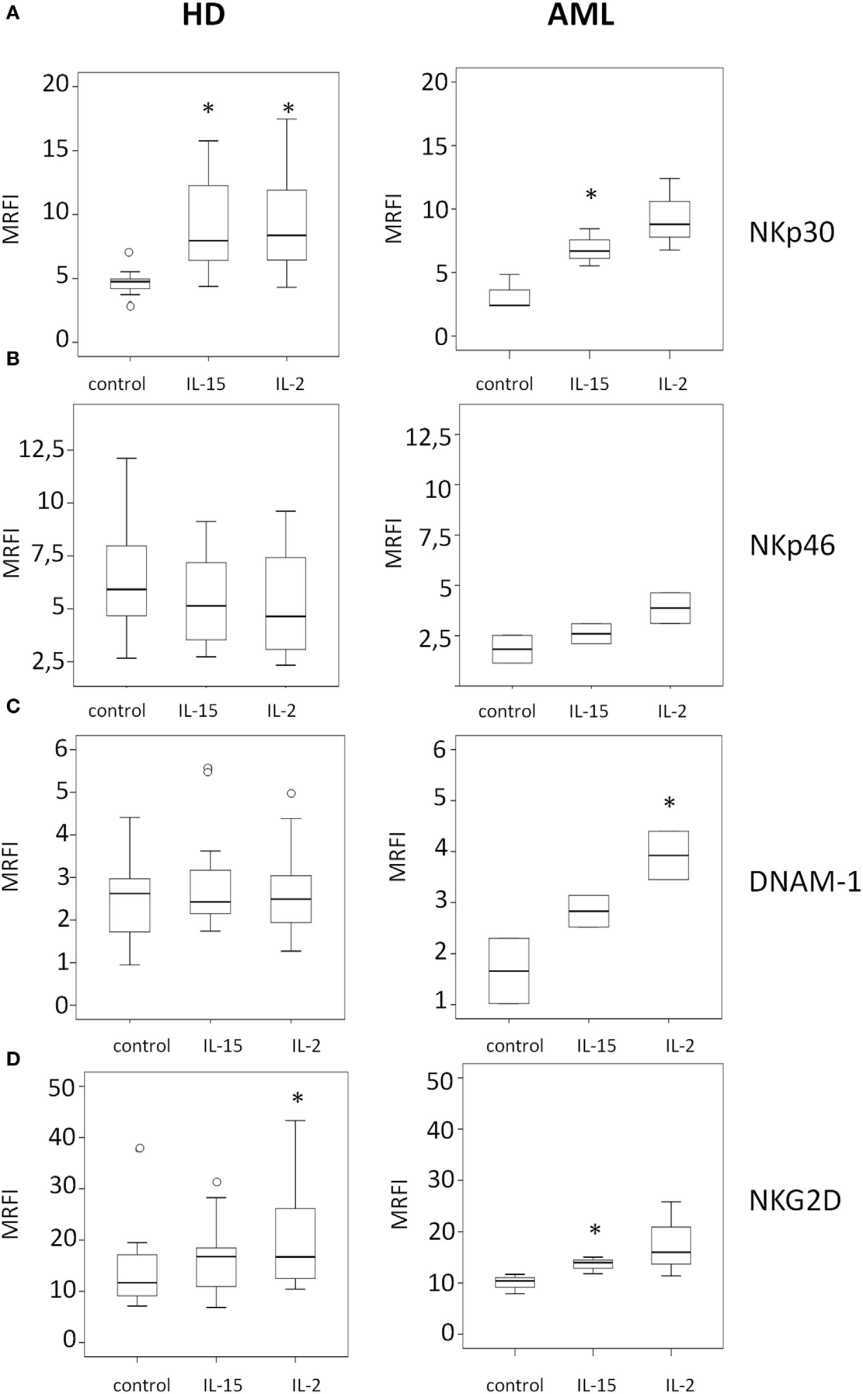
Modulatory effect of interleukin (IL)-15 and IL-2 on natural killer cell activating receptor expression in healthy donors and acute myeloid leukemia patients. The expression of NKp30 **(A)**, NKp46 **(B)**, DNAM-1 **(C)**, and NKG2D **(D)** was analyzed by flow cytometry. NK cell receptor expression was analyzed after 48 h of culture with medium alone (control), IL-15, or IL-2. Left panels represent HDs and right panels represent AML patients. Mean relative fluorescence intensity (MRFI) was calculated by dividing the mean fluorescent intensity (MFI) of the relevant monoclonal antibody by the MFI of its isotype control. The lower boundary of the box indicates the 25th percentile and the upper boundary the 75th percentile. Bars above and below the box indicate the 90th and 10th percentiles. The line within the box marks the median. Circles (○) represent outliers values. (*p < 0.05, compared to control).

In HDs (Figure 2, left panels), we observed a statistically significant upregulation of the NK cell-activating receptors NKp30 and NKG2D after culture of PBMCs with IL-2 (*p* = 0.004 and *p* = 0.013, respectively) (Figures 2A,D). IL-15 also induced a significant increase in the expression of NKp30 on NK cells surface (*p* = 0.0044) (Figure 2A), but the upregulation of NKG2D induced by IL-15 was not statistically significant (*p* = 0.08) (Figure 2D). No significant changes in the expression of NKp46 and DNAM-1 were observed (Figures 2B,C).

The analysis of the effect of IL-2 on NK cells from AML patients (Figure 2, right panels) only showed a statistically significant upregulation of DNAM-1 expression (*p* = 0.046) (Figure 2C), without significantly affecting the expression of the other receptors considered. By contrast, the incubation with IL-15 induced a significant increase of NKp30 and NKG2D (*p* = 0.001 and *p* = 0.028) (Figures 2A,D). A representative example of the effect of IL-15 is shown in Figure S1 in Supplementary Material.

### Cytotoxic Activity of Cytokine-Stimulated PBMCs

**Figure 3:**
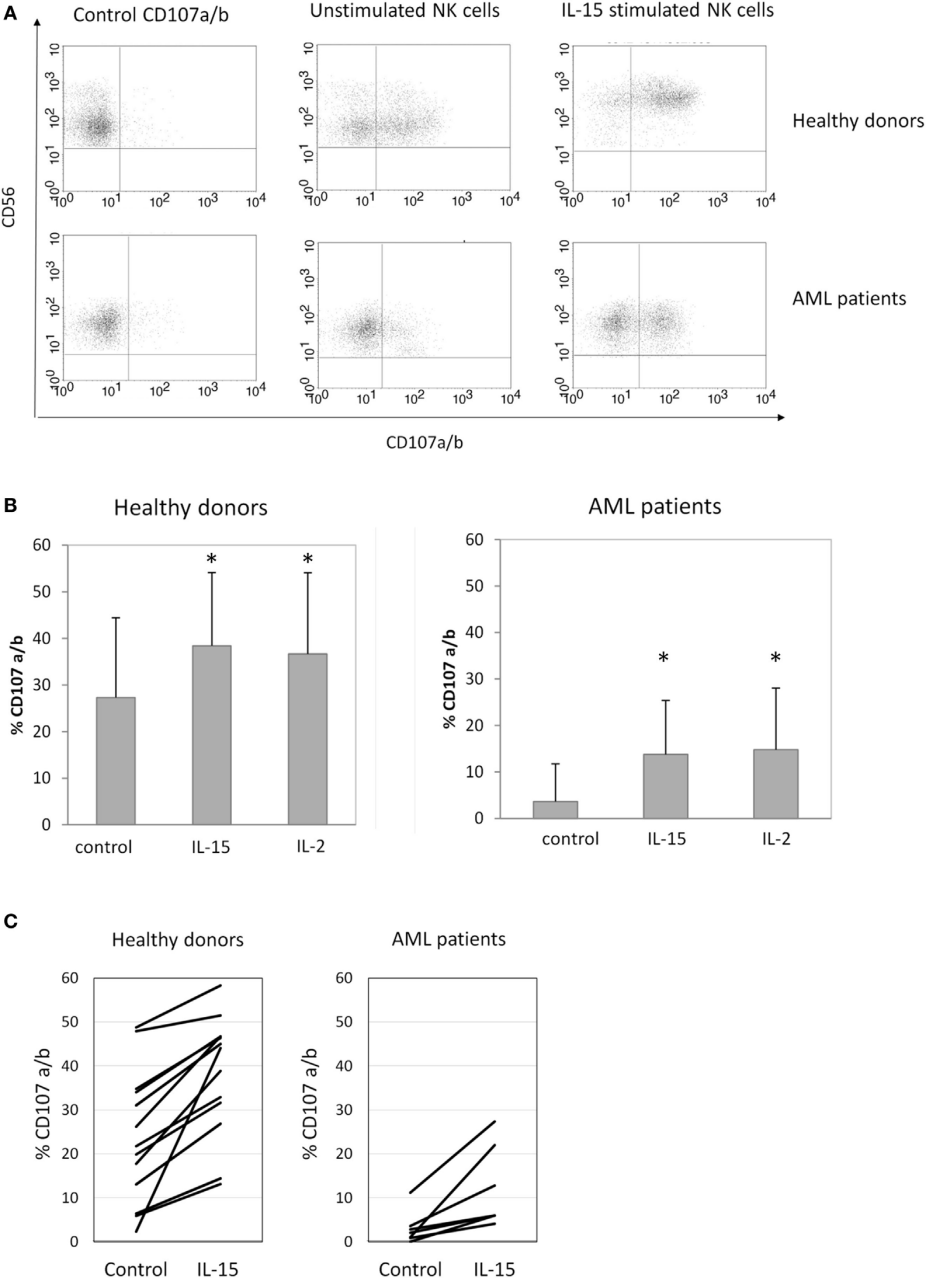
Natural killer (NK) cell degranulation after cytokine stimulation. **(A)** Representative flow cytometry analysis of NK cell degranulation against K562 cells. **(B)** Column bars show the percentage of CD107a/b expression on NK cells from healthy donors (HDs) (left graph, *n* = 16) or acute myeloid leukemia (AML) patients (right graph, *n* = 8) in response to K562 cells. Error bars represent SD. **(C)** Individual representation of NK cell degranulation against K562 cells in HDs (right graph) and AML patients (right graph). NK cells were used after 48 h of culture with medium alone (control), interleukin (IL)-15, or IL-2 (*p ≤ 0.05, compared to control).

Once we observed the effect of the different cytokines on NK cell receptor expression, we analyzed if these phenotypes correlated with the cytotoxic capacity of NK cells against K562, a susceptible target cell line. A representative example is shown in Figure 3A. Our results demonstrated that concomitant with the upregulation of the activating receptors following 48 h of IL-15 and IL-2 stimulation, there was a significant increase in NK cell cytotoxicity against K562 targets in both HDs and AML patients (Figure 3B). Although a high variability was observed, a positive effect of IL-15 on NK cell degranulation was found in all individuals analyzed but with different increases in degranulation (Figure 3C).

### Effect of IL-15 and IL-2-Stimulated PBMCs on Maturation of iDCs

Recent studies demonstrated that human NK cells display the ability to kill or induce maturation of both autologous and allogeneic monocyte-derived iDCs This function depends on the engagement of NKp30 with its cellular ligands expressed by DCs. Considering that IL-15 and IL-2 have a potent effect in the expression of NKp30 on NK cell surface, we decided to study if these modifications affect iDC maturation mediated by NK cells. iDCs were generated as described in Section “Materials and Methods,” harvested, counted and analyzed by FACS. After culture with cytokine-stimulated PBMCs, DC maturation was evaluated by the expression of CD86. Unstimulated PBMCs were used as control to compare the effect of IL-15 and IL-2 stimulation on PBMCs capacity to induce DC maturation. LPS-stimulated iDCs were used as positive control.

**Figure 4:**
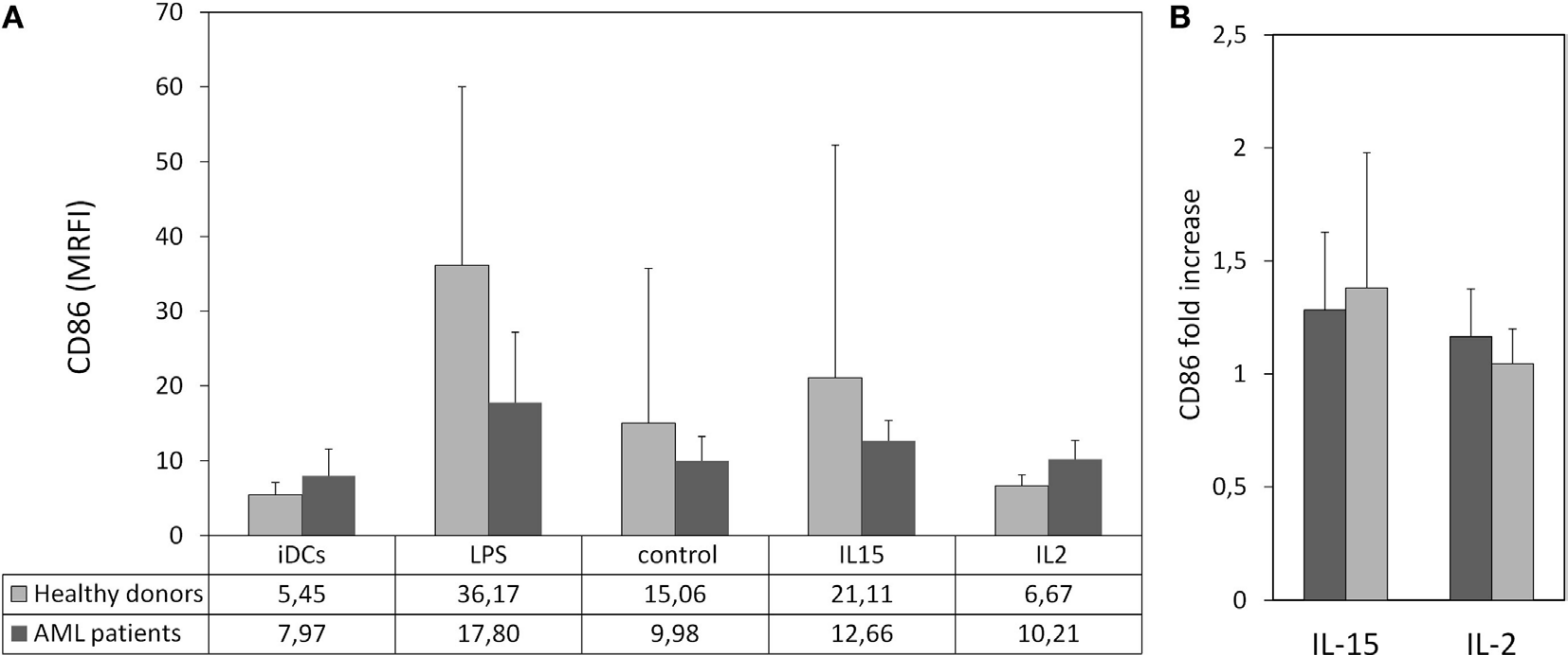
Maturation of immature DCs (iDCs) co-cultured with cytokine-stimulated peripheral blood mononuclear cell (PBMC). Allogeneic iDCs were cultured in the presence cytokine-stimulated PBMC from healthy donors (HDs) or acute myeloid leukemia (AML) patients. Lipopolysaccharide (LPS) was used as positive control. Dendritic cell (DC) maturation was characterized by the expression of CD86. **(A)** Column bars show the MRFI of CD86 expression on DCs from HDs after culture in the presence of LPS- or cytokine-stimulated PBMC from HDs (light gray, *n* = 6) or AML patients (dark gray, *n* = 4). PBMCs were used after 48 h of culture with medium alone (control), interleukin (IL)-15 or IL-2. Error bars represent SD. **(B)** Comparative analysis of the effect of cytokines on PBMC-mediated DC maturation. Bars represent fold increase of CD86 expression on DCs cultured with PBMC treated with IL-15 or IL-2 relative to the expression in DCs co-cultured with unstimulated PBMCs.

Our result showed that PBMCs from HDs and AML patients stimulated with IL-15 induced higher maturation of iDCs than unstimulated PBMCs although due to the high variability observed the differences were not statistically significant (Figure 4A; Figure S2). In response to cytokine-stimulated PBMCs, DCs displayed a onefold increase in the expression of CD86 compared to their response to unstimulated PBMCs (Figure 4B).

### Presence of IL-15 in Plasma of HDs and AML Patients

**Figure 5:**
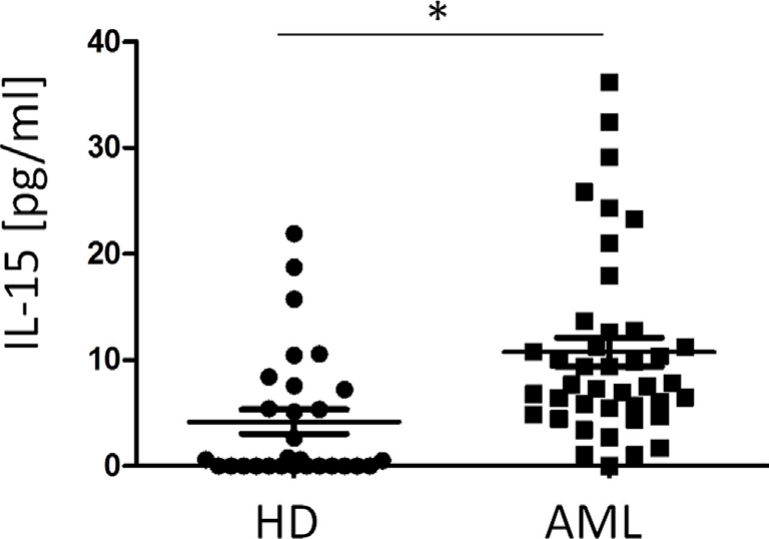
Comparison of plasma IL-15 concentration in healthy donors (HDs) and acute myeloid leukemia (AML) patients. The horizontal bars represent the mean values. *p ≤ 0.05.

Finally, we characterized the concentration of IL-15 in the plasma of HDs and AML patients. Our results showed that IL-15 was significantly higher in AML serum than in HDs (*p* < 0.001) (Figure 5). No correlation was observed between IL-15 levels in plasma and NKp30 expression on NK cells probably due to the high variability observed among donors (data not shown).

## Discussion

Acute myeloid leukemia is a heterogeneous disease that presents with different phenotypic and genotypic alterations in hematopoietic progenitors with the subsequent accumulation of immature hematopoietic stem cells that prevent the production of adequate amount of healthy hematopoietic cells. AML is more common in the elderly and co-morbidities and frailty often impact on patient tolerance to intensive treatment regimens. Thus, the prognosis of elderly AML patients remains poor, despite recent advances in the management and treatment options of AML patients including novel immunotherapies (50).

The role of NK cells against leukemia is supported by the discovery of NK cell spontaneous cytotoxicity against leukemia cell lines *in vitro* and the clinical benefits observed in KIR ligand mismatched allogeneic stem cell transplantation. Evidence for graft-versus-leukemia effect mediated by NK cells is observed in clinical studies with haploidentical donor transplants where the presence of alloreactivity due to KIR ligand mismatch was correlated with higher survival rates (51). NK cell-mediated cytotoxicity preserves healthy cells, and consequently, tumor control can be achieved in the absence of graft-versus-host disease (52). In addition, donor-derived NK cells have demonstrated to play a relevant role after hematopoietic stem cell transplantation (53).

Natural killer cells in AML patients habitually present defects in their cytotoxicity against autologous leukemic blasts probably as consequence of a reduced expression of activating receptors such as NCRs or DNAM-1 (36–38). Besides, the low expression of NKp46 and the NCR^dull^ (NKp46^dull^NKp30^dull^) phenotype have been associated with decreased survival in AML patients (38). NK cell phenotype and function at diagnosis of AML associate with clinical outcome. Thus, AML patients with low expression of activating receptors and decreased IFN-γ production had higher risk of relapse (54).

Previous reports demonstrated that IL-2 and IL-15 play an important role in the development, homeostasis, and function of T and NK cells. The administration of IL-2 as well as the adoptive transfer of IL-2-activated lymphocytes represented the first effective cancer immunotherapy for solid tumors such as melanoma. However, high doses of IL-2 were required to obtain therapeutic effect but a dose-related increase in toxicity was noted. Thus, IL-2 treatment was associated with adverse events including severe capillary leak syndrome that represent a major concern in IL-2 treatment. Besides IL-2 promotes the maintenance of Tregs that can reduce antitumor response (55). Since recombinant IL-2 was introduced, several clinical trials examining the role of IL-2 in preventing AML relapse have been developed. However, its use as monotherapy is not effective in terms of leukemia-free survival and overall survival (56). In a phase IV trial, AML patients in first complete remission received cycles of immunotherapy with histamine dihydrochloride and low dose of rIL-2 for 18 months to prevent leukemic relapse. The presence of high CD56^bright^ NK cell counts and high expression of NKp30 or NKp46 on CD16^+^CD56^+^ NK cells independently predicted leukemia-free survival and overall survival in this clinical trial (57).

Other cytokines have been proposed for cancer treatment, and, in the last years, IL-15 has emerged as a potential immunotherapeutic candidate (43). Preclinical studies suggest that IL-15 may represent a more efficacious cytokine for cancer immunotherapy with less toxicity obtained with intermittent administration of IL-15 compared with daily administration (45, 58).

We have analyzed *in vitro* the effect of IL-15 and IL-2 on NK cell phenotype and function including NK cell cross talk with DCs. In our study, IL-2 was included as positive control since its role on NK cell activation both *in vitro* and *in vivo* has been extensively reported. Recently, IL-15 has been proposed as an ideal candidate for the expansion of NK cells *in vivo* since it does not promote expansion of Tregs (59). In our study, IL-15 but not IL-2 induced a significant expansion of NK cells in HDs after 48 h of culture. By contrast, NK cell expansion was very limited in AML patients showing higher variability.

Our results also show that short time culture with IL-15 induces upregulation of NKp30 and NKG2D on NK cells from AML patients in concordance with previous reports (60). Other studies have also shown an increment of DNAM-1 and NKp46 expression after culture with IL-15 (40, 60). We have also observed an increment of DNAM-1 and NKp46 but this increase did not reach statistical significance. The upregulation of activating receptor expression induced by IL-15 is related to the increased NK cell degranulation against K562 cell line in concordance with previous reports (60, 61). Altogether, these results suggest that AML-induced defective function of NK cells could be overcome by IL-15. Besides, we detect a significant increased production of IFNγ and granzymes A and B after culture of NK cells with IL-15 (data not shown). These observations are consistent with previous reports supporting the pivotal role of IL-15 in NK cell antitumor activity (40, 60, 61).

Natural killer cell-mediated induction of DCs maturation was mediated by NKp30 and cytokines released after NK cell activation such as TNF-α and IFN-γ (62, 63). We have also found that IL-15-activated PBMCs from AML patients and HDs have a higher capacity to support maturation of DCs than untreated or IL-2 treated PBMCs, suggesting that the increased expression of NKp30 after IL-15 culture improves the capacity of NK cells to collaborate in the maturation of DC.

These results suggest that IL-15 in addition to enhance NK cell cytotoxicity could also collaborate with the development of adaptive immunity by promoting NK cell-mediated maturation of DCs. Further analysis will be required to confirm this function.

Cytokines present in the tumor microenvironment modulate antitumor responses. In AML patients, cytokine profile at diagnosis is frequently aberrant and associates with pathogenesis, disease progression, and survival. We have previously shown that plasma levels of TNF-α, IL-6, and IL-10 are increased in AML patients. Low levels of IL-6 and high levels of IL-10 were associated with longer event-free survival and patient survival (35). In the present work, since IL-15 has demonstrated a role in NK cell activation, we analyzed IL-15 plasma levels in AML patients. We found higher plasma IL-15 levels in AML patients compared to HDs confirming previous results (64). In lymphoid leukemia, it has been described an increase of IL-15 and IL-5R (65). It has been also shown that IL-15 could act as a growth factor for a minor fraction of AML cell lines expressing IL-2Rβ/γ promoting their survival and proliferation (66). In addition, the finding that AML patients have higher levels of IL-15 in plasma than HDs together with the possibility that IL-15 may promote blast growth has to be considered in protocols using IL-15 as adjuvant. We were surprised by the contradictory results showing high levels of plasma IL-15 in AML patients, whereas NKp30 expression on NK cells is diminished. It has recently been described that maintained levels of IL-15 may induce NK cell exhaustion (67), and it has been suggested that for *ex vivo* expansion optimal dosing and timing of IL-15 is critical to get adequate NK cell activation (68). We can hypothesize that maintained levels of IL-15 induce changes on NK cell phenotype (e.g., activating receptor expression) that further contribute to the diminished NK cell activity observed in AML patients.

Thus, in spite of the encouraging data of IL-15 immunotherapy in murine models, together with its low toxicity in mice and primates that has led to the design of clinical trials in AML patients, the use of IL-15 *in vivo* as monotherapy or combined therapy in AML patients requires special caution.

A better understanding of IL-15/IL15R axis will allow the identification of novel therapeutic strategies directed to increase IL-15 immunomodulatory effect contributing to antitumor immune response but avoiding the promotion of leukemic cells survival. Our *in vitro* results support the relevance of IL-15 to induce functional active NK cells in AML patients with enhanced capacity to destroy leukemic cells and induce DCs maturation.

## Ethics Statement

The study was approved by the local Ethics Committee and samples collected after written informed consent in accordance with the Declaration of Helsinki.

## Author Contributions

BS-C performed experiments. BS-C, CC, and AP analyzed data. JB, MJA, and HB selected the patients. BS-C, ED, RS, and RT designed the project and discussed data. BS-C and RT wrote the manuscript with support of all other co-authors.

## Conflict of Interest Statement

The authors declare that the research was conducted in the absence of any commercial or financial relationships that could be construed as a potential conflict of interest.
