# Supplementary figures and images for: In Vitro Culture with Interleukin-15 Leads to Expression of Activating Receptors and Recovery of Natural Killer Cell Function in Acute Myeloid Leukemia Patients
Source: Front Immunol. 2017 Aug 7;8:931. doi: 10.3389/fimmu.2017.00931 (PMC5545593; doi:10.3389/fimmu.2017.00931)

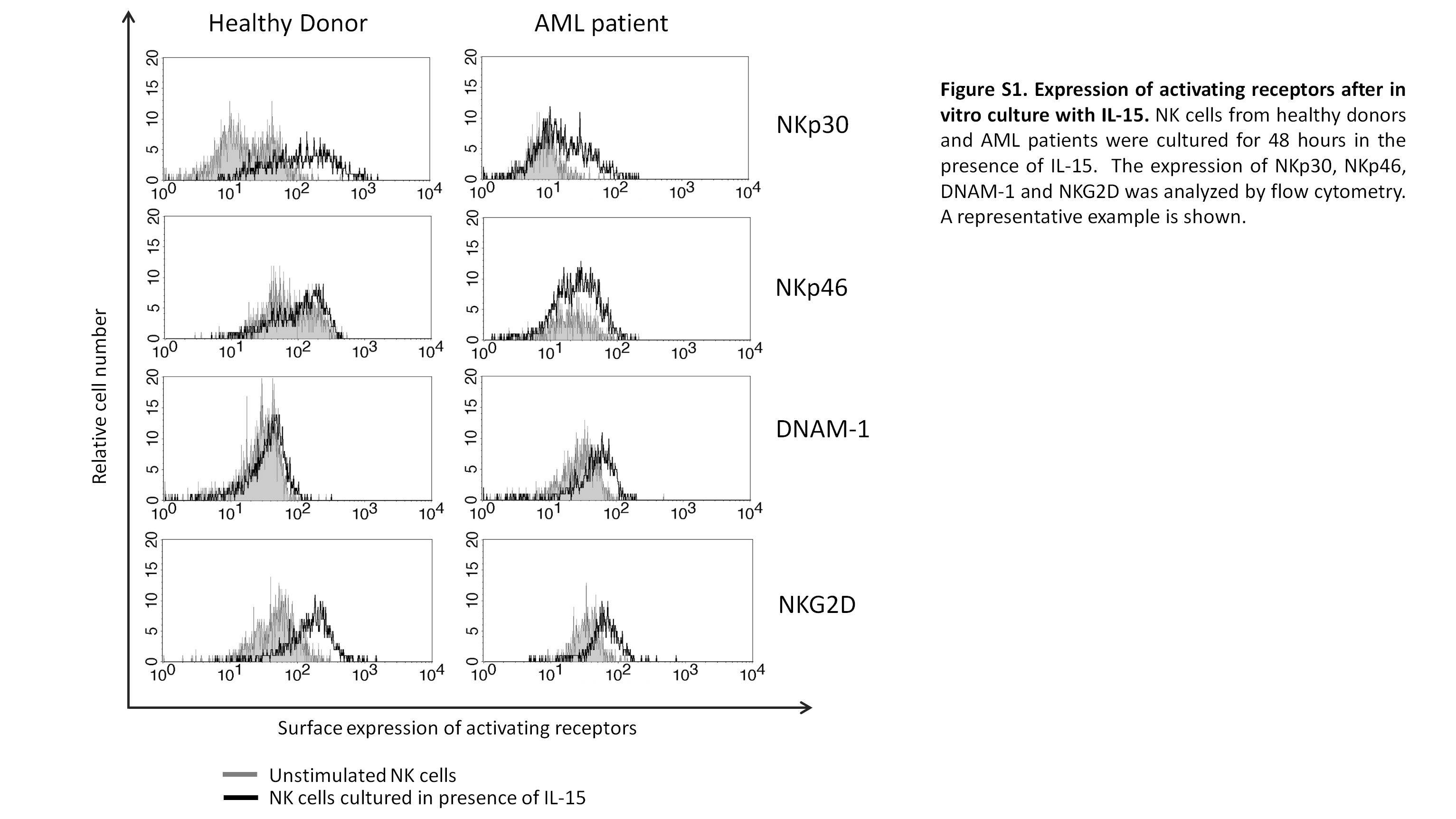

Supplement: Supplementary file 1 [file image_1.tif]

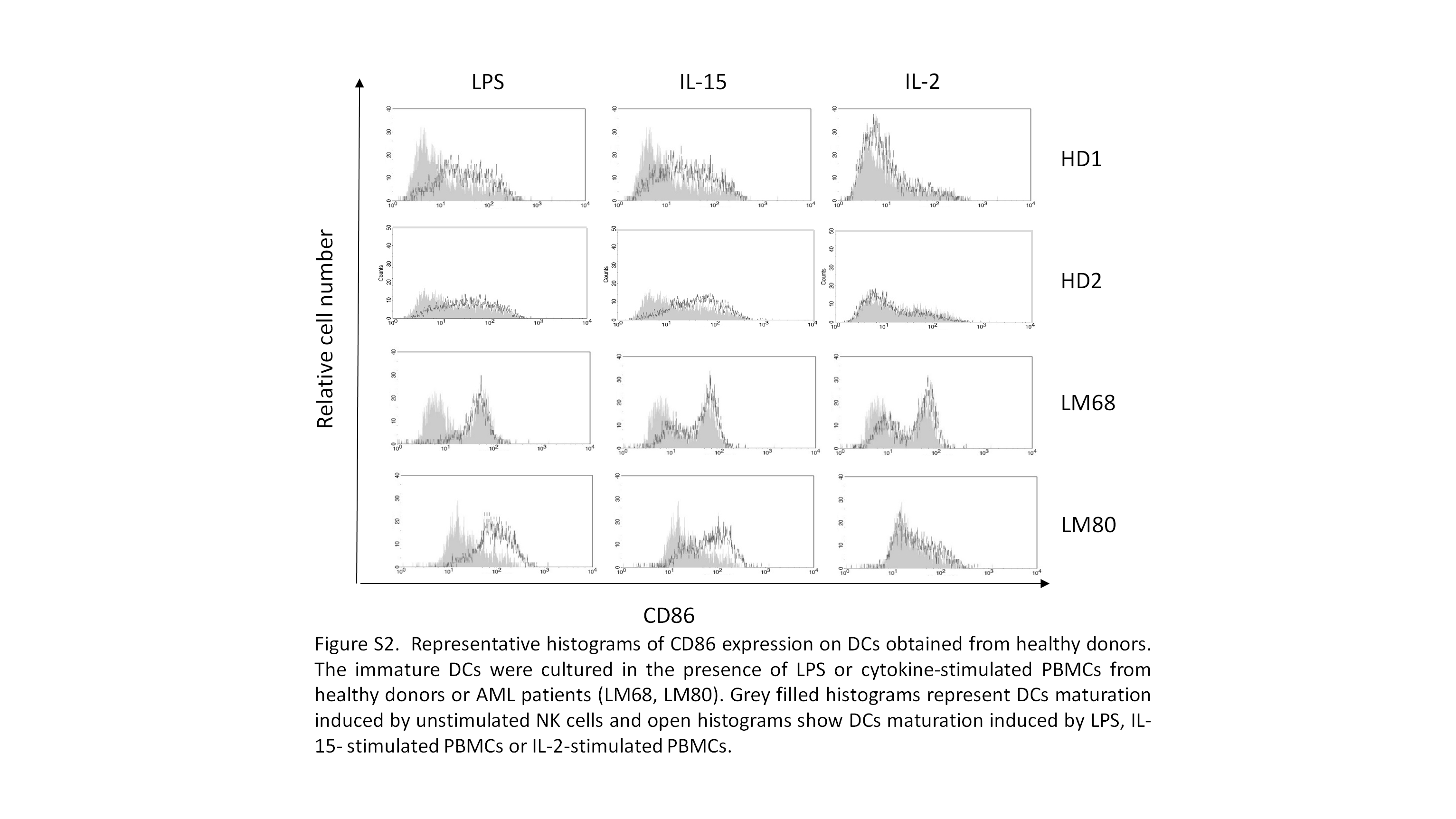

Supplement: Supplementary file 2 [file image_2.tif]
